# Fahr’s syndrome as a manifestation of autoimmune polyendocrine syndrome-1 and its unusual association with neuromyelitis optica spectrum disorder

**DOI:** 10.3389/fnins.2023.1226214

**Published:** 2023-06-30

**Authors:** Ahmad Nawaz, Azba Ahmad, Ayesha Aslam, Safia Bano, Ahsan Numan, Eisham Sarmad

**Affiliations:** Department of Neurology, Mayo Hospital, King Edward Medical University, Lahore, Pakistan

**Keywords:** Fahr’s syndrome, hypoparathyroidism, mucocutaneous candidiasis, autoimmune polyendocrine syndrome type-1 (APS-1), neuromyelitis optica spectrum disorder (NMO-SD)

## Abstract

Fahr’s syndrome, also known as bilateral striopallidodendate calcinosis, is a rare inherited neurodegenerative illness characterized by abnormal calcium deposition in several areas of the brain, resulting in a wide range of neuropsychological symptoms. Fahr’s syndrome, secondary to autoimmune polyendocrine syndrome type 1, which includes adrenal insufficiency and mucocutaneous candidiasis in addition to hypoparathyroidism, is exceedingly rare. No case report has been documented to date to show the co-occurrence of Fahr’s syndrome and neuromyelitis optica spectrum disorder. Here, we discuss the case of a 30-year-old man with a previous history of seizures and symptoms of ectodermal dystrophy presented with seizures, left-sided hemiparesis, dysarthria, and other characteristics indicative of severe hypocalcemia. The neuroimaging findings strongly suggested Fahr’s syndrome, with radiographic evidence of Neuromyelitis optica spectrum disorder as longitudinal extensive transverse myelitis in the cervical spinal cord, high titers of serum aquaporin-4 antibodies, and demyelinating neuropathy on nerve conduction studies. This distinct neuropsychological presentation and neuroimaging findings led to the diagnosis of Fahr’s syndrome as a result of hypoparathyroidism caused by autoimmune polyendocrine syndrome type 1 with cooccurrence of neuromyelitis optica spectrum disorder. The patient’s clinical symptoms improved considerably after he was treated based on a provisional diagnosis. The clinical importance of our case is significant for both neuropsychiatrists and endocrinologists, as autoimmune polyendocrine syndrome should be considered as the etiology of Fahr’s syndrome. This case report also aims to report this unusual association of Neuromyelitis optica spectrum disorder with Fahr’s syndrome to give the future prospective to know whether this association is incidental or there is a missing link between these two different disorders.

## Introduction

Fahr’s syndrome was first described by Karl Theodor Fahr in 1930. Fahr’s syndrome is a rare genetic disorder characterized by abnormal calcification of various areas of the brain, primarily the basal ganglia, thalamus, dentate nucleus of the cerebellum, frontal lobe, and temporal lobe, resulting in clinical neuropsychiatric manifestations including movement and gait disorders, cerebellar and speech abnormalities, and cognitive impairment (Zhou et al., 2019). Fahr’s disease is defined as primary familial idiopathic calcification with no underlying pathology, whereas Fahr’s syndrome is always caused by a secondary cause such as intragenic, genetic, infectious, or autoimmune disease that affects primarily the parathyroid gland resulting in hypoparathyroidism (Clarke et al., 2016; Perugula and Lippmann, 2016). Autoimmune polyendocrinopathy candidiasis ectodermal dystrophy (APECED), also referred to as autoimmune polyglandular syndrome type I (APS I), is a debilitating autosomal recessive condition characterized by a clinical trial of chronic mucocutaneous candidiasis, autoimmune hypoparathyroidism and Addison’s disease (Alkaphoury et al., 2015). Hypoparathyroidism is one of the most important clinical criteria required for the clinical diagnosis of Autoimmune polyendocrinopathy, candidiasis, and ectodermal dystrophy syndrome (APECED). Several case reports of cerebral calcification following hypoparathyroidism have been documented. However, no case has been reported to date that states APECED as the etiology of Fahr’s syndrome with other basic clinical characteristics of APECED in addition to hypoparathyroidism.

In this case report, we describe a patient with Fahr’s syndrome associated with hypoparathyroidism, persistent mucocutaneous candidiasis, and symptoms of ectodermal dystrophy who fits the criteria for APS type-1 with the co-occurrence of clinical and radiological manifestations of neuromyelitis optica spectrum disorder. During his stay, the patient was treated for neuromyelitis optica, manifestations of Fahr’s syndrome, and biochemical abnormalities, including hypocalcemia and hyperphosphatemia, which led to a dramatic improvement in his clinical condition.

## Case description

**Figure 1:**
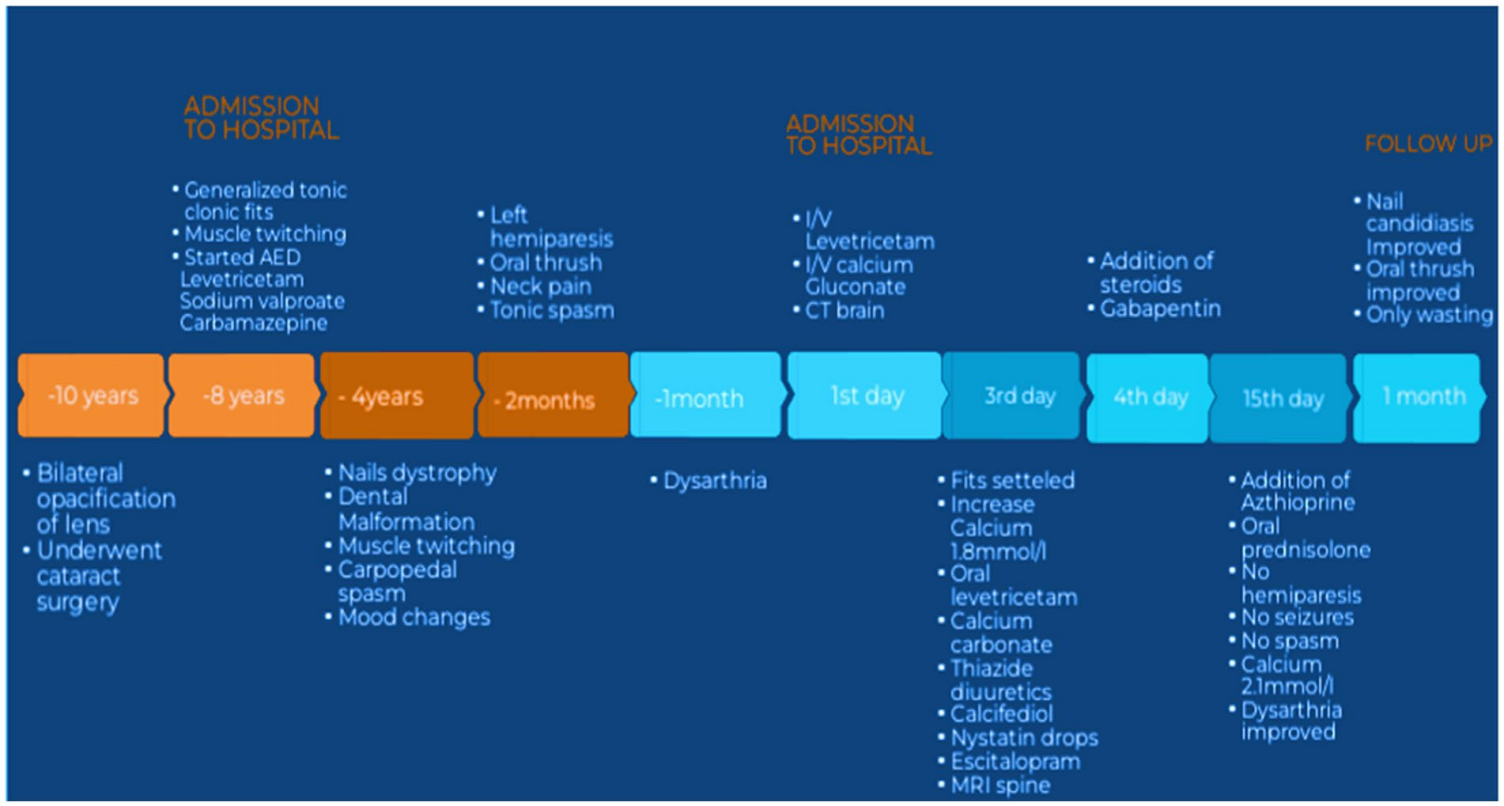
Showing timeline of events. AED, antiepileptic drugs; IV, intravenous; CT, computed tomography; MRI, magnetic resonance imaging.

A 30-year-old Pakistani man with known epilepsy was presented to the emergency department of a tertiary care hospital with complaints of more frequent generalized tonic–clonic fits (3–4 episodes per hour) for the last 3 h prior to his presentation. Fits were associated with up rolling of the eyes, urinary incontinence, tongue bite, and frothing from the mouth. The duration of each epileptic episode was between 2 and 3 min and was associated with post-ictal disorientation and confusion. On his admission to the neurology unit from the emergency department, he had severe, uncontrollable, spontaneous twitching of the muscles of his hands and feet that lasted for a very brief period of time. The patient was previously normotensive, non-diabetic, and non-alcoholic, with no history of prolonged fever or headache, head trauma, or cerebrovascular damage. For around 8 years, he had been treated for epilepsy with carbamazepine (400 mg twice a day), sodium valproate (500 mg twice a day), and levetiracetam (500 mg twice a day) with good compliance. There was a history of gradual onset, progressive left-sided hemiparesis for 2 months, affecting the left hand, which progressed to the left arm and leg within 15 days, with wasting of a small muscle of hand. The patient reported dysarthric speech that began within 30 days after the development of hemiparesis. Patients also report painful deglutition owing to oral thrush for around 2–3 months. On further inquiry, the patient additionally complained of severe, sudden shock-like pain radiating to both arms and throughout the back that worsened with neck movement in either direction and was also associated with numbness in both hands. There was also a complaint of painful spasm involving his arms and hands followed by abnormal flexed posturing of both arms, which usually occurred more frequently at night and lasted for more than 5 min, which disturbed the patient’s sleep and usually made him cry. He denied any concomitant blurred or double vision, pain, restriction in eye movements, or any other sensory complaints or sphincteric involvement. However, there was a history of perioral numbness and crawling sensation in both feet. There is no history of respiratory or gastrointestinal complaints, immunization, or oral ulcers; small or large joint pain; massive hair loss; or increased sensitivity to light or weight gain or weight loss, heat intolerance or cold intolerance or diarrhea but on and off constipation. He had never experienced such a weakness before. There was a history of increasing aggressiveness, mood instability, and irritability but no history of hallucinations, delusions, or cognitive deterioration. For 4 years, there had been a history of tooth deformity, aberrant coloring, and thickening of nails, all of which suggested ectodermal dystrophy, and he did not receive any treatment for it (Figure 1). He had undergone phacoemulsification surgery for cataracts in both eyes 10 years ago. There was no history of such complaints in any of his family members.

### Physical examination

**Figure 2:**
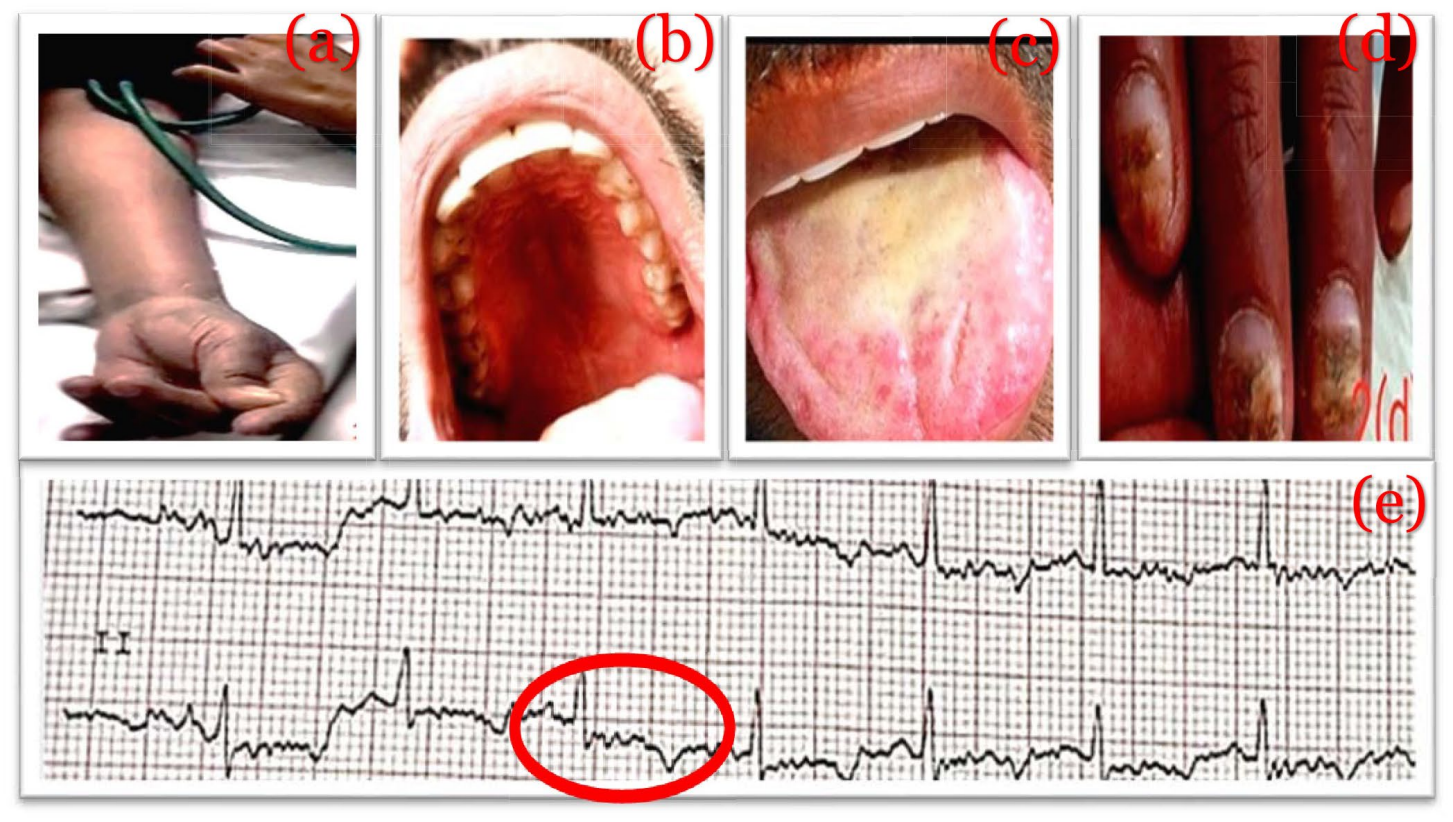
Sign of hypocalcemia and Ectodermal dystrophy. Chvostek’s sign **(A)**, maligned teeth **(B)**, oral thrush **(C)**, dystrophic nails **(D)**, ECG showing long QT of 0.46 s **(E)**.

On general physical examination, the patient was afebrile and hemodynamically stable. However, while taking blood pressure, abduction of the thumb, bending of the wrist and metacarpopharyngeal joint, and extension of the interphalangeal joints were observed, indicating tetanic carpopedal spasm, also known as Chvostek’s sign (Figure 2A). Subsequently, trousseau’s sign was also observed. Examination of the oral cavity revealed abnormally misaligned teeth and oral thrush (Figures 2B,C). Fingernails and toenails were dystrophic and malformed, with minor nail fold swelling (Figure 2D). During an ophthalmologic exam, pseudophakia was seen in both eyes. With normal extraocular movement of the eye, normal color perception, and contrast sensitivity, his visual acuity was 6/6 in both eyes. Pupils were equally reactive to light bilaterally with no afferent pupillary defect. The rest of the neurological examination in the neurology department revealed wasting of small muscles in the hands with decreased power (MRC grade: 4+) on the left side upper and lower limbs with slightly increased spastic tone (Modified Ashworth grade: 3) and almost normal tone and power on the right side. While biceps, triceps, knee, and ankle reflexes were absent on both sides and there was a bilateral extensor plantar response. Examination of the sensory system of the upper limbs revealed reduced pinprick sensation in the dermatological distribution of the upper limbs. The rest of the evaluation of the sensory system and cranial system was unremarkable. Signs of meningeal irritation were absent but Lhermitte’s sign was positive.

### Investigations

All routine baseline investigations were within normal limits, except for electrolytes. At the time of presentation, corrected serum calcium was reduced to 0.97 mmoL/L (normal: 2.2–2.6 mmoL/L), whereas serum phosphate was elevated to 1.97 mmoL/L (normal: 0.97–1.45 mmoL/L). The Vitamin D3 level was mildly decreased to 23.25 ng/dL (normal range: 30–100 ng/dL). The parathyroid hormone level was reduced to 4 pg./dL (normal range: 15.1–68.3 pg./dL). Aldosterone, HbA1c, and thyroid hormone levels were all in the normal range. ECG revealed a prolonged QTc of 0.44 s (Figure 2E).

**Figure 3:**
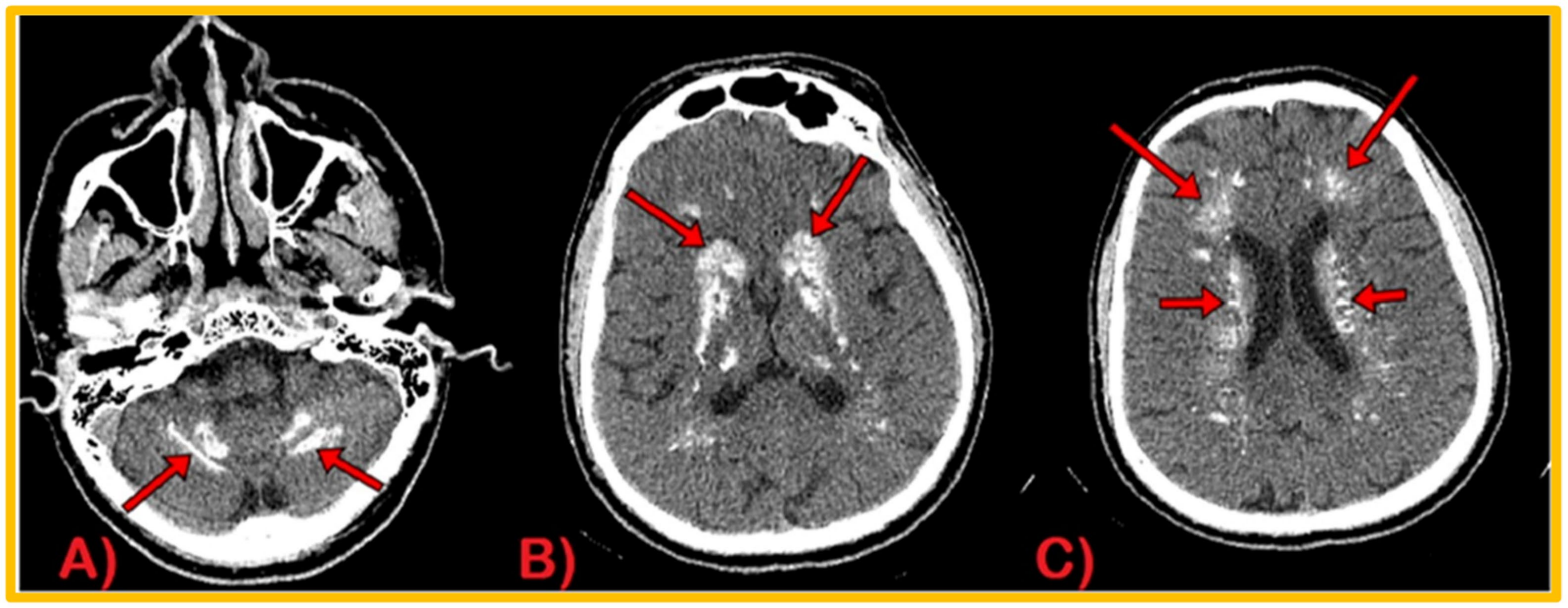
CT Brain Plain Axial views: calcification in dentate nuclei **(A)**, basal ganglia **(B)**, and periventricular and semiovale regions **(C)**.

**Figure 4:**
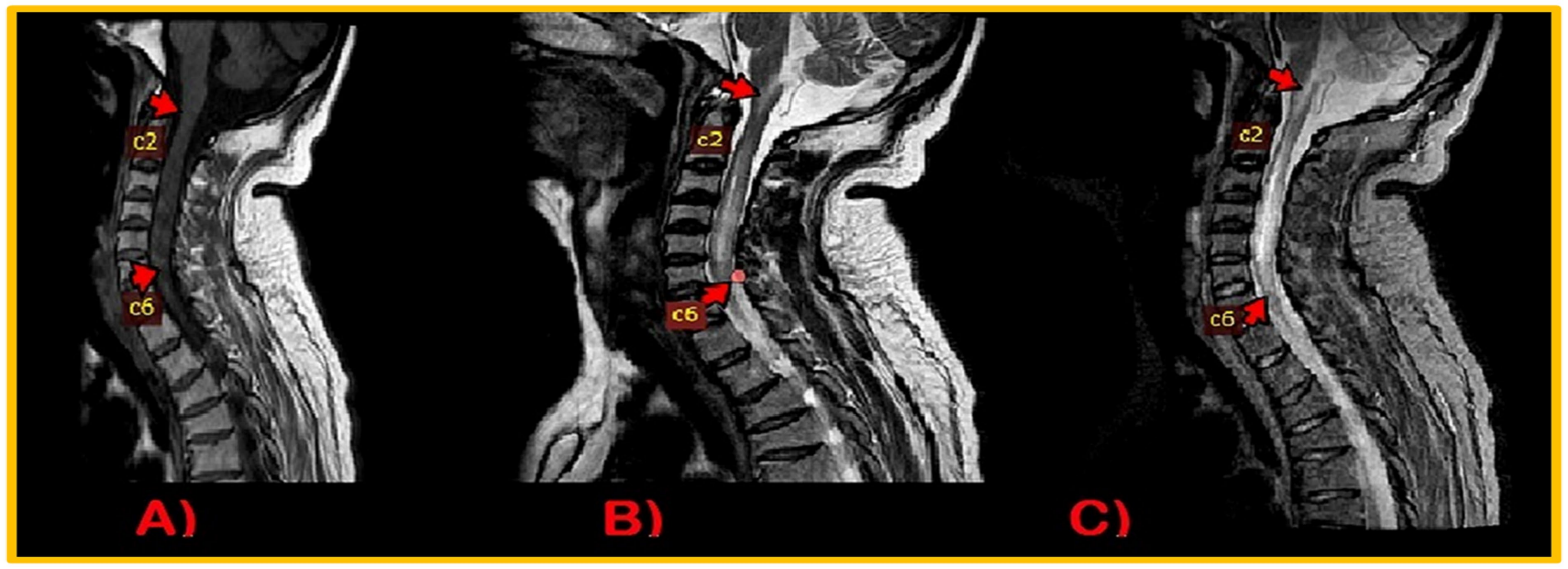
MRI Cervical Spinal cord: intramedullary altered intensity signals extending from craniocervical junction to C6 vertebral. T1WI, isointense **(A)**; T2WI/STIR, heterogenous hyperintense **(B,C)**.

During the recording, EEG showed no interictal epileptiform discharge or focal slowing. Nerve conduction studies revealed demyelinating polyneuropathy, both sensory and motor, primarily affecting the lower limbs. Candida species were cultured from nail scrapings. CT scan brain plain revealed symmetrical extensive calcification in the bilateral basal ganglia, dentate nuclei, periventricular, and semiovale regions of the brain (Figure 3). Neuroimaging showed altered t1w1 hypointense and T2 w1 hyperintense signal changes extending from the craniocervical junction to C6, causing diffuse cord expansion, suggesting acute longitudinal and extensive transverse myelitis (Figure 4). Serum AQP-4 antibody titers were around 120 U/mL using the ELISA technique. CT chest, abdomen pelvis done to rule out metastatic disease-causing brain calcification, was unremarkable. Additionally, a DEXA scan revealed age-inappropriately low bone mineral density due to increased bone turnover caused by hypoparathyroidism.

### Diagnostic approach and differential diagnosis

1. Initially, at the time of presentation, the patient was in status epilepticus with features of hypocalcemia (constipation, twitching of muscles, Chvostek’s sign, Trousseau’s sign, prolonged QTC) and no history of missed antiepileptic drugs or fever, So, the serum electrolyte imbalance (hypocalcemia) leading to metabolic seizures was considered.
2. The fact that the patient had hemiparesis for 2 months before the seizures started makes it necessary to consider scar epilepsy as a possible cause.
3. A history of epilepsy that was controlled with broad-spectrum antiepileptic drugs, and a current presentation of seizures, left-sided progressive hemiparesis, and dysarthria led us to consider associated paraneoplastic disease, autoimmune vasculitis (SLE), and metabolic cause (Fahr’s syndrome) as possible diagnoses.
4. A paraneoplastic cause was ruled out because the CT scans of the chest, abdomen, and pelvis were all normal and there was no history of illness that pointed to malignancy.
5. Autoimmune vasculitis was also ruled out because there were no signs of vasculitis and the ANA and ENA tests were normal.
6. Fahr’s syndrome was diagnosed based on the patient’s presentation, calcification of the brain on a CT scan, hypocalcemia secondary to hypoparathyroidism, and a negative workup for a paraneoplastic cause and autoimmune disease.
7. Low PTH levels, as well as biochemical and clinical evidence of hypocalcemia, mucocutaneous candidiasis, abnormally misaligned teeth, and discolored nails, led us to suspect APS-1(APECED) as the cause of Fahr’s syndrome.
8. In addition, the patient’s clinical history of progressive hemiparesis, dysarthria, painful spasms of the hands with diminished pinprick sensation, and bilateral extensor plantar response leads to a plan for neuroimaging for cord pathology in addition to the diagnosis of Fahr’s syndrome. Finally, by combining imaging with elevated CSF protein and high AQP-4 antibodies titers, we were able to establish the diagnosis of neuromyelitis optica spectrum disorder.

Due to the clinical symptoms of these two separate illnesses happening concurrently, the diagnosis of this case proved to be quite difficult. However, the clinical history, physical examination, and neuroimaging enable us differentiate these two conditions and manage the patient’s clinical symptoms properly.

### Treatment

Management strategies and treatment primarily focused on symptomatic relief targeting seizures, hypocalcemia and related clinical features, mucocutaneous candidiasis, and neuromyelitis Optica spectrum disease. In the emergency department, the patient was given 2 cc of diazepam diluted in 5 cc of normal saline slowly intravenously over 5 min, followed by a 3,000 mg intravenous levetiracetam infusion over 20 min. Correction of acute hypocalcemia was done by administering 2 g of calcium gluconate intravenously, followed by 10 g of calcium gluconate intravenously over 24 h. After 48 h in the neurology unit, the following oral medications were prescribed: 500 mg, levetiracetam orally twice a day, 1,000 mg of calcium carbonate three times a day, along with 12.5 mg of hydrochlorothiazide and 20 mg of calcifediol once a day, with continuous monitoring of calcium phosphate levels every 8 h and an ECG every 4 h. For oral thrush, Nystatin oral drops were given three times a day. The dermatology unit recommended 250 mg of oral terbinafine for 6 weeks to treat nail candidiasis. After consultation with the psychiatry department, the patient was also given an escitalopram 10 mg tablet to take by mouth once a day at night. The patient was given 1 g of intravenous methylprednisolone at the 4th day of his admission once a day for 5 days to treat neuromyelitis optica. Oral gabapentin of 75 mg once daily at night was added in the treatment regime for neuropathic pain. After completion of pulse therapy with methyl prednisolone for 5 days, he was given a tapering dose of equivalent oral prednisolone twice a day and 50 mg of oral azathioprine once a day. The patient responded to the treatment regimen. During his stay in the neurology ward, no clinical seizure activity, carpopedal spasm, or tonic spasm was noticed. After five doses of methylprednisolone, his hemiparesis and dysarthria also improved. While oral candidiasis and onychomycosis did not improve. His corrected calcium level increased to 2.12 mmoL/L. The patient was discharged 15 days after his admission to the hospital. It was advised to follow up with a repeat complete blood picture, liver function test, serum calcium level after 1 month, and parathyroid hormone level and vitamin D3 test after 3 months. It was decided that neurological imaging of the cord would only be scheduled in the event of any new neurological deficit.

### Follow-up and outcome

At the one-month follow-up visit, no new neurological deficit was noted. The corrected serum calcium level was 2.2 mmoL/L, while the corrected serum phosphorus level was 1.45 mmoL/L. Neither a full blood count nor liver function testing revealed any concerns. Additionally, oral thrush improved, and the condition of the nails significantly improved. Nystatin drops were stopped. Dermatology unit also opted to discontinue oral terbinafine and recommended to improve nail hygiene. The psychiatric ward recommended deep relaxation technique and continued escitalopram use, with monthly follow-ups. The patient reported ongoing paresthesia and numbness in his hands. Gabapentin dosage was increased to 100 mg once a day at night for this reason. As oral prednisolone was tapered to stop after 1 month, the patient was instructed to continue taking azathioprine and levetiracetam. Due to financial restrictions, the patient declined to repeat the parathyroid hormone or vitamin D level.

## Discussion

Fahr’s disease, Fahr’s syndrome, and striopallidodendate calcinosis all refer to idiopathic non-arteriosclerotic cerebral calcification of the striopallidodendate system. The familial or sporadic syndrome manifests as a variety of clinical symptoms, including pyramidal, extrapyramidal, and cerebellar signs with gradual cognitive deterioration. Moskowitz et al. outlined the diagnostic criteria for Fahr’s syndrome, which include neuropsychiatric manifestations and bilateral basal ganglia calcifications evident on neuroimaging in the absence of an infectious, toxic, or traumatic origin, as well as biochemical abnormalities and somatic signs suggestive of a mitochondrial, metabolic, or other systemic disease (Moskowitz et al., 1971; Ellie et al., 1989; Manyam, 2005; Donzuso et al., 2019). In our case, the patient had basal ganglia calcification on a CT scan, presenting with tetanic spasms, seizures, neuropsychiatric symptoms with underlying endocrine and autoimmune etiology. Hyperphosphatemia and hypocalcemia along with low parathyroid hormone values supported the suspicion of primary hypoparathyroidism. The most common causes of hypoparathyroidism include autoimmune diseases and neck surgery (Clarke et al., 2016). Autoimmune polyendocrine syndrome type is the rarest autoimmune cause of hypoparathyroidism. In addition to hypoparathyroidism, our patient had a history of nail dystrophy, maligned teeth, and oral mucocutaneous candidiasis, thereby fulfilling the APECED criteria and supporting that APS-1 is the underlying etiology of Fahr’s syndrome (Capalbo et al., 2013).

APECED syndrome, also known as autoimmunity polyendocrine syndrome type 1, is a rare autoimmune condition. APECED is characterized by a highly varied pattern of devastating autoimmunity that is primarily driven by specific autoantibodies directed against various endocrine and non-endocrine organs (Capalbo et al., 2013). The clinical diagnosis is established by the presence of two of the three classic components: chronic mucocutaneous candidiasis (CMC), chronic hypoparathyroidism (CH), and Addison’s disease (AD). Before the age of 10 years, chronic mucocutaneous candidiasis is often the first component to occur, followed frequently by chronic hypoparathyroidism and then adrenal insufficiency (Betterle et al., 1998). In addition to the major components, ectodermal dystrophy and additional endocrinopathies such as hypergonadotropic hypogonadism, insulin-dependent diabetes, autoimmune thyroiditis, and pituitary dysfunction may occur. Furthermore, gastrointestinal illnesses (chronic atrophic gastritis, pernicious anemia, malabsorption, autoimmune hepatitis, and cholelithiasis), skin diseases (vitiligo and alopecia), keratoconjunctivitis, immunological abnormalities, and asplenia may be present (Betterle et al., 1998; Capalbo et al., 2013). Immune-mediated central and peripheral neurological manifestations, including chronic inflammatory demyelinating polyneuropathy and posterior reversible encephalopathy syndrome, are rarer consequences of the condition (Meloni et al., 2012). However, no cases of Fahr’s syndrome or APS-1 in association with neuromyelitis optica spectrum disorder have been documented. Progressive hemiparesis, tonic upper limb spasm, diminished pinprick sensation in both upper limbs, bilateral extensor plantar response, and a positive Lhermitte’s sign helped to localize the lesion in the cervical cord in our patient. Findings of longitudinal extensive transverse myelitis with positive aquaporin-4 antibodies confirmed the diagnosis of neuromyelitis optica. Furthermore, difference in the grading of reflexes in the upper and lower limbs were extremely misleading for us. It was assumed that the patient also had an underlying neuropathy, which was later validated by nerve conduction studies and electromyography.

It is extremely unusual for Fahr’s syndrome and APS-1 to co-occur with the neurological manifestation of neuromyelitis optica spectrum disorder. NMO can occur independently of Fahr’s syndrome, and the combination of the two disorders can be accidental in this scenario. On the one hand, these two facts can be used to explain their co-occurrence:

1. Fahr’s syndrome is primarily a neurodegenerative disorder; damage to the blood–brain barrier and neurons in this syndrome results in the release of inflammatory mediators in these cells as well as an increase in the production of proinflammatory cytokines like IL-6 and the stimulation of T helper (Th) 17 cells (Abedini et al., 2015). As a result, a subset of B-cells in the peripheral tissues are activated to produce anti-AQP4 IgG antibodies. The BBB’s disruption in Fahr’s syndrome allows auto-reactive B cells to penetrate and undergo class switching to create IgM autoantibodies. This antibody binds complement to astrocytic foot processes expressing AQP4 antigen. Complement-dependent cytotoxicity and complement-dependent cellular cytotoxicity both cause harm to stellate astrocytes. Bystander inflammatory injury or, more likely, the loss of trophic support from astrocytes may have an impact on oligodendrocytes that are proximal to astrocyte foot processes. The resulting demyelination causes loss of saltatory conduction and conduction block, which leads to neurological deficits (Kinoshita et al., 2010; Bukhari et al., 2012).
2. It is also possible to hypothesize that the deletion of the AIRE gene in APECED causes T helper cells (Th) to escape self-tolerance, release proinflammatory cytokines that can trigger inflammation, and activate auto-reactive B cells, which in turn produces autoantibodies including that cause central nervous tissue inflammation and damage. The majority of APECED components can also be linked to the formation of certain autoantibodies (Ramsey et al., 2006; Kisand et al., 2010; De Martino et al., 2013).

Finally, it can be concluded, it is unclear which pathogenic mechanism contributes to Fahr’s disease and NMO coexisting. However, when two diseases occur in the same patient, each with an unknown cause, it is necessary to investigate their likely link. Research is recommended about the concurrence of NMO with brain calcification. Moreover, the robust response of clinical manifestations to steroids and other immunosuppressive agents also strengthens our hypothesis of a single factor of autoimmunity. There are some limitations that the authors would like to point out, such as the fact that they cannot conduct genetic studies or cytokine testing like IL-6 to confirm the proposed mechanism. Moreover, follow-up sessions could not be completed due to the patient’s poor visits and financial constraints.

## Conclusion

Fahr’s syndrome secondary to hypoparathyroidism has not been previously documented in any patient with APECED. In this article, we discussed the extremely rare syndrome APECED (APS-1) as the cause of Fahr’s syndrome. The clinical significance of our case is essential for neuropsychiatrists and endocrinologists to early identify this uncommon cause of Fahr’s syndrome and emphasize the multidisciplinary approach to this syndrome. The purpose of this case report is also to draw attention to the unique relationship between Neuromyelitis optica spectrum disorder and Fahr’s syndrome, as well as to shed light on the possible pathogenesis of co-occurring between these two disorders. This case also encourages researchers to investigate the missing link behind the occurrence of these two distinct disorders together.

### Patient’s perspective

I have been experiencing severe, disabling spasm in my arms that has made me cry and disturbed my sleep. I have been using polypharmacy for my seizures. I had consulted many physicians and psychiatrists for my mood problems. But the root cause could not be figured out. After additional tests, I am now aware of the cause of my disease and I’m happy for the quick recovery of my left-sided weakness; my seizures are controlled with only one drug; my nails look better cosmetically; and I am now having an enjoyable life.

## Author contributions

AAh made substantial contributions to the conception, design, and acquisition of data. ANa involved in drafting the manuscript and revising it critically for important intellectual content. AAs and SB involved in actively management of the patient and contributed to the analysis and interpretation of data. ANu given final approval to the version to be published. All authors contributed to the article and approved the submitted version.

## Conflict of interest

The authors declare that the research was conducted in the absence of any commercial or financial relationships that could be construed as a potential conflict of interest.

## Publisher’s note

All claims expressed in this article are solely those of the authors and do not necessarily represent those of their affiliated organizations, or those of the publisher, the editors and the reviewers. Any product that may be evaluated in this article, or claim that may be made by its manufacturer, is not guaranteed or endorsed by the publisher.
